# Patients with a Higher Number of Periodic Limb Movements Have Higher Nocturnal Blood Pressure

**DOI:** 10.3390/jcm11102829

**Published:** 2022-05-17

**Authors:** Klaudia Krzyzaniak, Eemil Partinen, Markku Partinen, Mariusz Sieminski

**Affiliations:** 1 Emergency Department, University Clinical Centre, Smoluchowskiego 17, 80-214 Gdansk, Poland; 2 Departament of Neurological Sciences, University of Helsinki, 00100 Helsinki, Finland; eemil.partinen@gmail.com; 3 Vitalmed Research Center, Helsinki Sleep Clinic, Valimotie 21, 00380 Helsinki, Finland; 4 Helsinki Sleep Clinic, Terveystalo Healthcare, Department of Neurosciences, Clinicum, University of Helsinki, 00100 Helsinki, Finland; markku.partinen@terveystalo.com; 5 Department of Emergency Medicine, Medical University of Gdanski, Smoluchowskiego 14, 80-214 Gdansk, Poland; mariusz.sieminski@gumed.edu.pl

**Keywords:** periodic limb movements, blood pressure, polysomnographic

## Abstract

There is growing evidence that periodic limb movements in sleep (PLMS) may lead to increased blood pressure (BP) values during the night. The aim of this study was to assess if patients with disordered sleep and an increased number of PLMS have higher BP values at night. We analyzed 100 polysomnographic (PSG) recordings of patients with disordered sleep, with the exclusion of sleep-related breathing disorders. Patients also registered beat-to-beat blood pressure during PSG. We compared the BP of patients with an increased number of PLMS (more than 5 PLMS per hour of sleep) during the night (examined group, *n* = 50) to the BP of patients with a PLMS number within the normal range (up to 5 PLMS per hour of sleep) (control group, *n* = 50). Patients from the examined group had significantly higher values of systolic BP during the night (119.7 mmHg vs. 113.3 mmHg, *p* = 0.04), sleep (119.0 mmHg vs. 113.3 mmHg, *p* = 0.04), and wake (122.5 mmHg vs. 117.2 mmHg, *p* = 0.04) periods and of diastolic BP during the night (75.5 mmHg vs. 70.6 mmHg, *p* = 0.04) and wake (77.6 mmHg vs. 71.5 mmHg, *p* = 0.01) periods. Our results suggest a relationship between the number of PLMS during the night and the values of nocturnal blood pressure. It is possible that their treatment could lower nocturnal BP in patients with sleep disorders, therefore improving their vascular risk profile.

## 1. Introduction

There is a constantly growing interest in the clinical significance of nocturnal blood pressure (BP). It is well established that both a lack of a nocturnal dip in the values of BP and isolated nocturnal hypertension increase the risk of cardiovascular events and of target organ damage [1,2,3,4].

Low quality of sleep and any sleep disturbances may lead to significant increases in nocturnal BP values, as was shown in previous studies [5,6]. Therefore, it should be examined as to which sleep-disturbing conditions are related to increased values of nocturnal blood pressure, with special attention given to the curable ones. An example of the latter can be periodic limb movements in sleep (PLMS). PLMS are sleep-related involuntary movements, characterized by repetitive flexions of the toes, ankles, knees and hips. They are defined as leg movements lasting from 0.5 to 10 s, occurring in a series of at least four movements with intervals between single movements lasting from 5 to 90 s. PLMS may be associated with transient cortical activation observed in EEG recording—in such a situation, the term PLMS with arousal (PLMS-A) is used. The EEG changes related to PLMS have diffuse scalp distribution being mostly concentrated in motor and sensory cortical areas [7]. PLMS-As appear in most patients with PLMS, although its frequency and causes of differences in its frequency among subjects were not analyzed so far. PLMS are found in the vast majority of patients with restless legs syndrome (RLS), but they are also frequently recorded in subjects suffering from narcolepsy, REM behavior disorder or idiopathic hypertension, as well as in healthy subjects [8,9,10].

The frequency of PLMS is described with the PLMS index (PLMS-I): the number of PLMS per hour of sleep. Five PLMS per hour (PLMS-I = 5) is considered as the upper limit of the norm [9].

There are studies describing a transient significant increase in BP values following each PLMS, both in patients with RLS and in healthy subjects [11,12,13]. This increase was higher in PLMS with associated arousals (PLMS-A). It was also shown that a higher number of PLMS leads to higher values of nocturnal blood pressure in patients with insomnia [14]. The hypothetic relationship between the number of PLMS and nocturnal blood pressure is of special interest, as PLMS can be successfully suppressed with pharmacological treatment. Therefore, the aim of this study was to verify the hypothesis that patients with disordered sleep with an increased number of PLMS have higher values of nocturnal blood pressure.

## 2. Materials and Methods

The protocol for the study was approved by the Independent Bioethical Committee for Scientific Research at the Medical University of Gdansk.

### 2.1. Subject

We retrospectively analyzed all the polysomnographic (PSG) recordings performed in the tertiary sleep clinic from January 2011 to February 2012. We analyzed recordings of all the patients requiring diagnostic procedures due to problems with initiating or maintaining sleep. Patients with sleep-related breathing disorders (SRBD, defined as apnea/hypopnea index (AHI) > 5) were excluded from the study. All included patients—if treated for any reason—that had a stable drug regime for at least 2 weeks prior to PSG. From the remaining patients, we selected all subjects with a periodic limb movement in sleep index (PLMS-I) ≥ 5 as the examined group. Subjects with PLMS-I < 5 constituted the control group.

### 2.2. Recordings

Patients underwent a single polysomnographic study. All the recordings were performed with the SOMNOscreen plus PSG system (Somnomedics, Randersacker, Germany). Sleep recording included four EEG leads, two bilateral electro-oculogram leads (EOG), bilateral chin electromyographic (EMG) leads, and two surface EMG leads placed on the left and right anterior tibialis muscles (recording PLMs in sleep and wake). Respiration was recorded with a nasal cannula, thoracic and abdominal strains and finger 2 oximetry. ECG was recorded with a single precardial lead. PSG also included BP measurement.

BP measurements were non-invasive, to avoid additional sleep disturbance. The measurement was based upon pulse transit time (PTT). A finger 2 photoplethysmograph was attached to the patient’s finger. Its role was to detect pulse waves. The time between detection of the R-wave on the ECG and the detection of the corresponding pulse wave was measured, and then, using a previously described mathematical model [15], the value of blood pressure was calculated. This method was recently successfully validated against cuff-based measurements [16]. It was also used in CPAP-using patients and in sleep-disordered patients [17,18]. The BP measuring system was calibrated at the beginning of recording by a single auscultatory BP measurement. The auscultatory BP measurement was then repeated at the end of the recording.

### 2.3. Analysis of the Recordings

Standards of the American Academy of Sleep Medicine (AASM) were used for sleep scoring [19]. PLMS were scored according to World Association of Sleep Medicine (WASM) standards [8]. Arousals were scored according to AASM rules.

Mean blood pressure values were measured for the following periods: sleep, wake, day and night. The “sleep” and “wake” periods were defined according to PSG recordings in concordance with AASM standards, and “day” was defined as the time from the beginning of the recording until the “light off” moment. This was a 30 min-long period, with patients staying awake before switching the light off. “Night” was defined as the period between the “light off” and “light on” moment.

### 2.4. Statistical Analysis

The normality of the distribution of different variables was tested using the Shapiro–Wilk normality test. All of the parametrical data were compared with a one-way t-test. The non-parametrical data were compared with a chi-square test. The reliability of multiple comparisons regarding values of blood pressures in both groups was checked with the Bernoulli formula to assess the probability of accidentally significant results [20].

## 3. Results

There were 50 subjects in the examined and control groups. The demographic and clinical data of the patients are presented in Table 1. Cardiovascular disease was defined as the presence of coronary heart disease, cerebrovascular disease or peripheral artery disease in the patients’ history.

Sleep parameters of the examined and control groups are shown in Table 2. There was a significant difference in PLMS-I and PLMSA-I, resulting from inclusion in the examined and control group. As PLMSA-I is proportional to global number of PLMS, it was also significantly higher. The group also differed in terms of sleep efficiency, wake index (number of awakening per hour of sleep), arousal index (number of cortical arousals per hour of sleep) and in time spent in sleep stage 1 and in REM sleep.

The mean values of systolic and diastolic blood pressure during sleep, wake, night and day were higher in the examined group, with significant difference for systolic BP in sleep, wake and night and for diastolic BP in wake and night (Table 3). The score of the Bernoulli formula was 0.00001, suggesting very low likelihood of accidental significance. Average heart rate was higher in the examined group, although the difference did not reach statistical significance (61.02 beats per minute vs. 59.32 beats per minute; *p* = 0.3).

## 4. Discussion

Analyzing PSG recordings, we found that patients with increased PLMS-I (above 5) have higher values of systolic and diastolic blood pressure compared to subjects with PLMS-I within the normal range (below 5).

Discussing these results, we would like to seek explanations of this result, define its clinical significance and propose its practical implications.

There are three possible explanations for the observed difference in the blood pressure values. The first explanation, and the most intuitive one, relies on the observation that each periodic limb movement is followed by an increase in values of systolic and diastolic blood pressure. This phenomenon was noticed first by Ali [21]. Then, it was thoroughly examined in studies applying beat-to-beat blood pressure measurements. Pennestri, analyzing recordings of patients with restless legs syndrome, found an increase of (average) 18 mmHg in SBP and of 9 mmHg in DBP following PLMS [12]. The same authors also observed this phenomenon in healthy (not suffering from RLS) subjects—the increase in SBP and DBP following PLMS was smaller than in patients with RLS but still significant compared to the baseline [11]. Siddiqui et al. also observed a transient increase in BP following PLMS, with a significant (11.2 mmHg) increase in SBP and a noticeable but not significant increase in DBP [13]. Recently, Cassel et al. found that PLMS are related to most of the increases in nocturnal BP in patients with RLS [22]. These results suggest that PLMS trigger a sympathetic activation leading to an increase in BP. The simplest explanation for our results, in the context of the studies cited above, is that subjects with a larger number of PLMS (in our study, it was over 20-fold larger) experienced very frequent transient significant increases in their BP, resulting in increased nocturnal blood pressure.

Further studies focusing on the analysis of cardiovascular events surrounding PLMS allowed for another hypothesis to be proposed that explains the higher BP values in subjects with higher PLMS-I. Ferillo et al. found that a significant change in EEG activity and heart rate is detectable just prior to the PLMS [23]. Similar results were described by Ferri et al., showing an increase in EEG activity and in heart rate immediately preceding PLMS [24]. Sasai et al., analyzing heart rate variability (HRV), found that periods of sleep with the appearance of PLMS are preceded with an increase in sympathetic nervous system activity [25]. These results suggest that an increase in sympathetic tone preceding PLMS may exist and, therefore, PLMS could instead be one of the effects of sympathetic activation (together with BP and HR increase) and not a trigger of changes in cardiovascular functions. It can be hypothesized then that subjects with a higher number of PLMS have a higher level of sympathetic activity, which may result in higher nocturnal values of BP.

The third explanation could be that the increased values of BP were a consequence of disordered sleep. Our data showed that patients with higher PLMS-I had lower sleep efficiency and a higher number of awakenings. It was found by Au et al. that diminished sleep efficiency and sleep duration are related to increased values of blood pressure [26]. Similar findings were reported by Javaheri [27]. Experimental deprivation of slow-wave sleep led to increases in nocturnal blood pressure [28]. The following sequence of events may be hypothesized to explain our results: the presence of PLMS leads to derangements in sleep architecture, resulting in an increase in nocturnal blood pressure.

Studies on relations between PLMS and blood pressure focused on analysis of the sleep microarchitecture. A significant amount of data was collected showing noticeable activation of the vascular system following an isolated movement of the leg. The novelty of our study is that we have analyzed the global impact of PLMS on nocturnal blood pressure. Our results, showing that a higher number of PLMS is related to increased blood pressure throughout the night, suggest that PLMS therapy may reduce BP and protect patients against the consequences of increased BP or hypertension.

Our study has some limitations. We focused on BP in sleep-disordered patients, as this was the aim of the study. Nevertheless, adding a control group of subjects with undisturbed sleep would add more data to explain the observed phenomenon. Nevertheless, healthy controls with normal sleep parameters would not help in determining which sleep parameter is the crucial one for increasing the values of BP. It must also be noted that PTT-based BP measurement, although trustworthy and frequently used, is still not validated in night-long studies. As our recordings were performed under the constant control of a technician, and the BP measuring system was carefully calibrated before the recording, we consider the measurements used in our study reliable.

There is a growing body of evidence suggesting existing links between PLMS (and RLS) and cardiovascular morbidity. Batool-Anwar et al. observed that RLS is related to risk of hypertension [29]. Further studies showed that patients with RLS have non-dipping patterns of blood pressure [6,30]. PLMS were found to increase the risk of cardiovascular morbidity and hypertension [31,32]. PLMS may also be linked with targeted organ damage, as they were shown to lead to left ventricular hypertrophy by Mirza et al. [33]. It must be noted that PLMS can be successfully treated, e.g., with dopaminergic drugs [34], and it was shown that such a therapy may normalize the cardiac reaction to PLMS [35]. Therefore, our results suggest that PLMS may be considered as a risk for increased values of nocturnal blood pressure, and as it is a treatable phenomenon, it may be considered as a modifiable vascular risk factor.

## Figures and Tables

**Table 1:** Demographic and clinical data of the patients.

Variable	Examined Group (*n* = 50)	Control Group (*n* = 50)	*p*
Age (mean ± SD **; years)	46.0 ± 15.1	40.6 ± 14.3	0.07
Gender (men/women)	23/27	23/27	1.00
BMI * (mean ± SD; kg/m^2^)	26.7 ± 4.6	24.9 ± 4.6	0.62
Arterial hypertension (*n;* %)	12; 24%	9; 18%	0.52
Cardiovascular disease (*n*; %)	7; 14%	4; 8%	0.52
Diabetes mellitus (*n*; %)	2; 4%	0; 0%	0.49
Cardiac arrhythmia (*n*; %)	4; 8%	3; 6%	1.0
Depression (*n*; %)	14;28%	14; 28%	1.0
Insomnia (*n*; %)	27; 54%	31; 62%	0.54
RLS *** (*n*; %)	16; 32%	5; 10%	0.01
Narcolepsy (*n*; %)	1; 2%	1; 2%	1.0
Daytime somnolence	13; 26%	6; 12%	0.124

* BMI—body mass index; ** SD—standard deviation; *** RLS—restless legs syndrome.

**Table 2:** Sleep parameters of the examined and control groups.

Parameters	Examined Group	Control Group	*p*
TST * (minutes, mean ± SD)	382.7 ± 71.9	409.4 ± 72.7	0.07
Sleep Efficiency (%)	76.5 ± 10.8	81.3 ± 11.3	0.03
Sleep Latency (minutes, mean ± SD)	30.6 ± 33.4	25.7 ± 28.7	0.44
Sleep stage change index	18.9 ± 5.3	16.9 ± 5.8	0.08
WASO ** (minutes, mean ± SD)	81.1 ± 50.4	63.8 ± 48.8	0.09
Wake Index	5.7 ± 2.6	4.2 ± 2.6	0.01
S1%	18.8 ± 8.2	14.2 ± 8.1	0.01
S2%	47.4 ± 8.3	61.6 ± 87.1	0.25
SWS%	16.9 ± 8.3	15.9 ± 7.9	0.53
REM% ***	17.0 ± 6.4	20.1 ± 7.0	0.02
AHI ****	1.4 ± 1.3	1.4 ± 1.2	0.96
PLMS-I *****	26.5 ± 19.8	1.0 ± 1.5	<0.00005
PLMSA-I ******	5.2 ± 4.8	0.1 ± 0.3	<0.00005
Arousal Index	18.4 ± 8.5	12.5 ± 5.7	0.0001

* TST—total sleep time; ** WASO—wake after sleep onset; S1%—% of sleep spent in S1 sleep stage; S2%—% of sleep spent in S2 sleep stage; S3%—% of sleep spent in S3 sleep stage; *** REM%—% of sleep spent in REM sleep stage; **** AHI—apnea/hypopnea index; ***** PLMS-I—periodic limb movements in sleep index; ****** PLMSA-I—periodic limb movements in sleep with arousal index.

**Table 3:** Mean values of systolic and diastolic blood pressure in sleep, wake, night and day.

Parameters	Examined Group	Control Group	*p*
SBP in sleep (mean ± SD; mmHg)	119.0 ± 14.6	113.3 ± 13.2	**0.04**
SBP in night ± SD (mean ± SD; mmHg)	119.7 ± 15.0	113.8 ± 12.9	**0.04**
SBP in day (mean ± SD; mmHg)	126.6 ± 14.9	120.3 ± 11.9	0.05
SBP in wake (mean ± SD; mmHg)	122.5 ± 13.7	117.2 ± 21.5	**0.04**
DBP in sleep (mean ± SD; mmHg)	75.2 ± 10.9	71.3 ± 9.2	0.06
DBP in night (mean ± SD; mmHg)	75.5 ± 10.9	70.6 ± 13.0	**0.04**
DBP in day (mean ± SD; mmHg)	77.9 ± 10.8	74,1 ± 9.4	0.06
DBP in wake (mean ± SD; mmHg)	77.6 ± 10.2	71.5 ± 14.1	**0.01**

## Data Availability

Not applicable.
